# Open-Label Placebo Trial among Japanese Patients with Chronic Low Back Pain

**DOI:** 10.1155/2020/6636979

**Published:** 2020-12-28

**Authors:** Tatsunori Ikemoto, Takefumi Ueno, Young-Chang Arai, Norimitsu Wakao, Atsuhiko Hirasawa, Kazuhiro Hayashi, Masataka Deie

**Affiliations:** ^1^Department of Orthopedic Surgery, Aichi Medical University, Nagakute, Japan; ^2^Division of Clinical Research, National Hospital Organization Hizen Psychiatric Center, Saga, Japan; ^3^Institute of Physical Fitness, Sports Medicine and Rehabilitation, Aichi Medical University, Nagakute, Japan; ^4^Multidisciplinary Pain Center, Aichi Medical University, Nagakute, Japan; ^5^Department of Orthopedic Surgery, National Center for Geriatrics and Gerontology, Obu, Japan

## Abstract

**Background:**

The aim of this study was to confirm the effectiveness of open-label placebo (OLP) in Japanese patients with chronic low back pain (CLBP), similar to previous reports, and to investigate its short- and medium-term effects in this study population.

**Methods:**

Fifty-two patients with CLBP were randomized into a treatment as usual (TAU) group (*n* = 26) or an OLP + TAU group (*n* = 26) for 12 weeks. The TAU included advice to remain active and exercise in conjunction with recent psychological education based on a self-management strategy. In contrast, participants in the OLP + TAU group were instructed to take two OLP capsules a day. Outcome measures were assessed at baseline and at weeks 3 and 12 using the Roland–Morris Disability Questionnaire (RMDQ), Numerical Rating Scale (NRS) for pain intensity, and the Timed-Up-and-Go (TUG) test. Difference in outcomes between the two groups was compared at the two follow-up points.

**Results:**

Although all participants completed the 3-week follow-up, four patients (two in each group) were lost to follow-up beyond week 3. There were no significant intergroup differences in changes in the RMDQ score (*p*=0.40), pain-NRS score (*p*=0.19), and TUG time (*p*=0.98) at week 3. Two-way repeated measure analyses of covariance showed significant time-course effects but did not show group effects or any interactions between the time-course and group in terms of the RMDQ score. However, it did not show any effects in the pain-NRS score and TUG time at week 12.

**Conclusions:**

The OLP + TAU group showed no superior findings in comparison with the TAU group after 3 weeks and 12 weeks for Japanese patients with CLBP. Nonetheless, significant improvements in functional disability were observed in both groups.

## 1. Introduction

Chronic low back pain (CLBP), usually defined as pain lasting or recurring for longer than 3 months, is the most common musculoskeletal problem globally [1, 2]. CLBP remains the leading global cause of activity limitation, absenteeism from work, and years lived with disability [3]. The practice guidelines for chronic pain management aim to optimize pain control as well as improve functional disability, recognizing that a pain-free state may not be attainable [3, 4].

Placebo medicines without any active ingredients have been widely used in clinical practice [5, 6]. The placebo pill is commonly used only in clinical trials for new medicines and is considered unethical as a therapeutic drug because its administration to a patient who is unaware of its pharmacological inactiveness is assumed to deceptive. Nevertheless, open-label placebo (OLP) has been recently reported to show therapeutic benefits for several clinical symptoms even when patients are aware that the medicine is a placebo [7]. Two studies suggested that OLPs with treatment as usual (TAU) for 3 weeks led to improvements in disability and reductions in pain intensity among patients with CLBP, in comparison with TAU only [8, 9]. These findings suggest that OLPs may provide important new treatment possibilities for CLBP across countries.

Notably, however, there remain unsolved methodological concerns that should be addressed to achieve general acceptance of OLP usage for CLBP [10]: (1) the control group (e.g., the TAU group) in clinical trials should be rigorously determined because the definition of usual care seems to be inconsistent across studies [11]; (2) there are contextual effects that include not only the placebo pill but also the physician's skill and the physician-patient relationship [12], implying that both OLP and OLP + TAU therapeutic interventions should be performed consistently by the same clinician; (3) trials of a longer duration are needed to explore the sustainability and long-term effects of OLPs [9]. In addition, recent studies suggest that potential racial differences exist in the effects of placebos [13, 14].

To address these concerns, the aim of this study was to confirm whether OLP would be as effective among Japanese patients with CLBP as in the report by Carvalho et al. and to investigate its short-term (3 weeks) and medium-term (12 weeks) effects on this sample by using patient-reported outcomes of functional disability.

## 2. Methods

### 2.1. Study Design

A randomized controlled trial design was established prior to this confirmatory study. We hypothesized that OLP + TAU would be superior to TAU in CLBP patients with an effect size of 0.74 in the RMDQ score based on previous findings [8]. On the basis of this assumption, the sample size for a power of > .80 and a two-tailed *α* at a significance level of < .05 was a minimum of 48 participants (24 per each arm). Finally, we recruited 52 participants (26 per each arm) after considering a 10% dropout rate. The trial was registered at the UMIN Clinical Trials Registry (UMIN000033412) on July 17, 2018. This study was approved by the Ethics Committee of our institution, and all study participants provided written informed consent.

### 2.2. Participants

Participants who were recruited consecutively at our tertiary center from August 2018 to March 2020 were included in the study. In order to avoid bias attributable to the patient-physician relationship, all candidates were new consultations to the principal investigator (T.I). The inclusion criteria were age over 20 years, persistent CLBP of a minimum duration of 6 months, and a score of at least 3 on a 10-point Numerical Rating Scale (NRS) for subjective pain intensity. The exclusion criteria were as follows: (1) no wish to participate, (2) pain radiating to the lower leg, (3) ongoing treatment for malignant tumor, spinal fractures, or spinal infections, (4) autoimmune diseases, (5) spondyloarthropathy, (6) age 75 years and older with a T-score lower than 2.5 on dual-energy X-ray absorptiometry, (7) history of fragile spinal fractures, (8) history or current use of steroids for more than 3 months, (9) prominent spinal kyphosis or degenerative lumber scoliosis (Cobb angle over 30°), (10) thoracic or lumbar surgeries performed less than 3 months previously, (11) medication associated with dementia, (12) suspected cognitive disorder, and (13) cases considered ineligible by the researchers. A total of 60 patients with CLBP were screened for study participation by an experienced orthopedic clinician (T.I), and 52 participants were enrolled.

### 2.3. Randomization and Intervention

The patients were randomized in a 1 : 1 ratio into TAU or OLP + TAU groups with stratification by age (less than 50 years old and over 50 years old) by using computer-generated random numbers that were inserted in numbered, opaque, and sealed envelopes. All treatments were performed by a single orthopedist (T.I) throughout the study because the levels of practitioner, confederate, and behaviors should be structurally equivalent [10]. Any prior medications used for the treatment of comorbidities, such as hypertension and diabetes, were unchanged.

Both groups received TAU for 3 months in accordance with recommendations from clinical practice guidelines [3]. The treatment included advice to remain active along with education and reassurance as first-line care. Moreover, a recently described psychological education based on a self-management strategy [15] was used to improve pain-related disabilities. When patients required second-line care, pharmacological treatments except new analgesic agents were allowed for both groups. At the baseline session, all participants underwent a one-hour session with the same orthopedist (T.I) for initializing this intervention.

The OLP + TAU group additionally received the OLP capsule with sufficient information. At baseline, the patients were provided standardized information of the placebo effect [8,9]. Briefly, this information covered “five points”: (1) the placebo effect is powerful; (2) the placebo effect has been confirmed in the previous literature; (3) the body can automatically respond to a placebo capsule like Pavlov's dogs which salivated when they heard a bell; (4) a positive attitude helps but is not necessary; and (5) taking the pills faithfully is critical. The patients were informed that the placebo capsules did not contain any active ingredients. Each OLP capsule contained 450 mg of lactose. The patients received 2 capsules orally twice (morning and evening) a day for 12 weeks. Capsule intake was recorded in a patient diary. Participants were regarded as dropouts when they failed to take more than 80% of the total prescribed capsules during the first 3 weeks or the next 9 weeks.

We investigated patterns of drug treatments that have been routinely used in usual care in both groups [16] to clear the contents of the TAU. No new medications were allowed in both groups, and only tapering of current medications was allowed during the 12 weeks.

### 2.4. Outcome Assessments

Outcome measures were assessed at baseline and at weeks 3 and 12 by using the Roland–Morris Disability Questionnaire (RMDQ), the Numerical Rating Scale (NRS) for pain intensity, and the Timed-Up-and-Go (TUG) test. The degree of subjective satisfaction was measured only in the OLP + TAU group.

The RMDQ was used to measure subjective disability caused by low back pain at each assessment [17], and the Japanese translated version of the RMDQ was used in this study [18]. The RMDQ score assessed the patients' disability caused by back problems, and the results were analyzed statistically. The RMDQ consisted of 24 items. The total RMDQ scores ranged from 0 to 24, with higher scores indicating higher levels of disability. Cronbach's *α* was 0.73 for the total RMDQ score.

The NRS was used to measure subjective pain severity, where 0 = no pain and 10 = worst pain imaginable [19].

The TUG test, a performance-based measure of physical functioning, is a measure frequently used to assess basic mobility [20]. This test has been used in a variety of patient groups, including those with low back pain [21]. The patients were given verbal instructions to rise up from a chair, walk 3 m as quickly and as safely as possible, cross a marked line on the floor, turn around, walk back to the chair, and sit down. All of these outcomes were measured by the same principal investigator (T.I).

Subjective satisfaction for overall improvement was determined at month 3 after baseline. Patient satisfaction was evaluated using a 5-point subjective satisfaction scale with the following options: “Very Satisfied,” “Somewhat Satisfied,” “Neither Satisfied nor Unsatisfied,” “Somewhat Unsatisfied,” or “Very Unsatisfied.”

### 2.5. Statistical Analysis

We initially tested if the data were normally distributed using the Shapiro–Wilk normality test for continuous variables. Continuous variables were represented as mean and standard deviation or medians and interquartile ranges (IQRs) in accordance with their distributions, while categorical variables were represented as the number and percentage of patients. Variables in each group at baseline were compared using chi-square test, Student's *t*-test, or Mann–Whitney *U* test.

A statistical testing strategy consisting of primary efficacy analysis (changes from baseline in the RMDQ score, NRS score, and TUG time after 3 weeks of treatment) as well as the secondary efficacy analyses (changes in the RMDQ score, NRS score, and TUG time after 12 weeks of treatment) was employed. The primary outcomes were compared using Student's *t*-test or Mann–Whitney *U* test, and effect size (Cohen's d).

Cohen's *d* refers to the standardized mean difference between two groups of independent observations and is given by the following equation:

(1) $$\begin{array}{c} \text{d}=\frac{\left(\mu_{1}-\mu_{2}\right)}{\sqrt{\left(n_{1}-1\right)s_{1}^{2}+\left(n_{2}-1\right)s_{2}^{2}}/\left(n_{1}+n_{2}-2\right)}. \end{array}$$

The secondary outcomes were analyzed using analysis of covariance (two-way ANCOVA) as covariates of age and sex, followed by Bonferroni comparison.

We also performed a responder analysis to compare characteristics of participants who achieved a minimal clinically important difference (MICD) in pain reduction and those who achieved a MICD in functional disability. The term “responder analysis” refers to the dichotomization of a continuous primary efficacy measure into “responder” and “nonresponder” categories [22]. MICD of pain reduction in Japanese individuals with CLBP was reported to be at least Δ2 after treatment [23]. Since MICD of patient-reported outcomes was known to be approximately half of the SD in a target outcome [24], we defined a RMDQ responder as the one that improved at least 0.5∗SD of baseline RMDQ scores in all participants. Success rates between the two groups at each follow-up point were then compared using a chi-square test.

The data were analyzed using SPSS (version 26.0 for Microsoft Windows; SPSS, Chicago, IL, USA). A *p* value < 0.05 was considered statistically significant.

## 3. Results

### 3.1. Patient Characteristics

**Figure 1:**
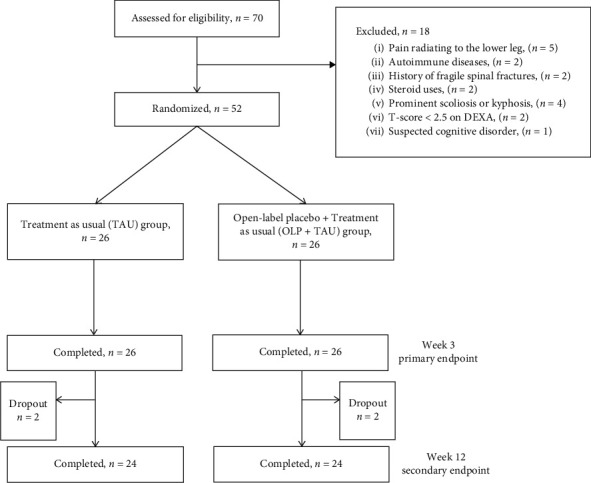
Flow diagram of the randomized trial comparing the TAU group or TAU + OLP group.

Between August 2018 and March 2020, 70 patients with CLBP were initially referred to the primary investigator, and 18 patients were excluded due to the eligibility criteria.  Figure 1 is a flowchart of participant disposition. In total, 52 consecutive patients with CLBP were randomized to the TAU or OLP + TAU groups. All participants completed the 3-week follow-up. Of the 52 patients, 2 patients in the TAU group were lost to follow-up beyond week 3 because of personal reasons, whereas 2 patients in the OLP + TAU group intentionally dropped out of the study after week 3. The remaining 48 patients (92.3%; 24 in each group) completed the 12-week follow-up.

Of the 52 patients, 32 (68.2%) were women and the mean age (±SD) was 66.8 (13.4) years. The mean values (±SD) of the RMDQ score, NRS score, and TUG time at baseline (*n* = 52) were 10.1 (±3.8), 5.4 (±1.7), and 8.8 (±2.2), respectively. No variables showed significant differences between the two groups at baseline (Table 1).

### 3.2. Changes in Outcome Values

**Figure 2:**
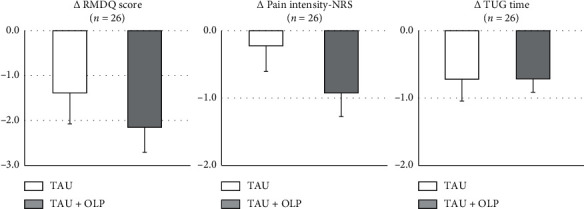
Changes in outcomes by the treatment group at week 3. Mean change scores on RMDQ score, pain-NRS, and TUG time. Error bars represent standard errors of the mean. RMDQ: Roland–Morris Disability Questionnaire; NRS: Numerical Rating Scale; TUG: Timed-Up-and-Go.

Figure 2 shows the changes from baseline in the RMDQ score, pain-NRS score, and TUG time by the treatment group at the primary endpoint. There were no significant intergroup differences in changes in the RMDQ score (*p*=0.40, *d* = 0.24), pain-NRS score (*p*=0.19, *d* = 0.38), and TUG time (*p*=0.98, *d* = 0.01) at week 3.

**Figure 3:**
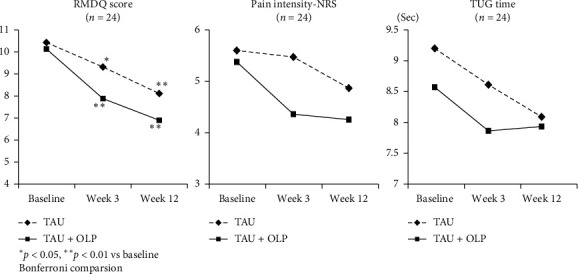
Changes in outcomes by the treatment group.

Table 2 and Figure 3 show the changes in the RMDQ score, NRS score, and TUG time by the treatment group at the secondary endpoint. Two-way repeated measure ANCOVA showed significant time-course effects (*F*_(2,42)_ = 4.65, *p*=0.02, *η*_*p*_^2^ = 0.18) but did not show group effects (*F*_(1,43)_ = 0.82, *p*=0.37, *η*_*p*_^2^ = 0.02) or any interactions (*F*_(2,42)_ = 0.72, *p*=0.49, *η*_*p*_^2^ = 0.03) between time-course and groups, as indicated by the RMDQ score. In contrast, when comparing NRS scores and TUG time, ANCOVA did not show any significant changes in the time-course (*F*_(2,42)_ = 1.83, *p*=0.17, *η*_*p*_^2^ = 0.08; *F*_(2,42)_ = 2.39, *p*=0.10, *η*_*p*_^2^ = 0.10), group effects (*F*_(1,43)_ = 1.89, *p*=0.18, *η*_*p*_^2^ = 0.04; *F*_(1,43)_ = 1.19, *p*=0.28, *η*_*p*_^2^ = 0.03), or interactions (*F*_(2,42)_ = 1.00, *p*=0.37, *η*_*p*_^2^ = 0.05; *F*_(2,42)_ = 1.06, *p*=0.36, *η*_*p*_^2^ = 0.05) between the time-course and group. Post hoc Bonferroni comparison revealed that patients in both groups showed significant improvement in functional disability at the 3-week and 12-week follow-ups.

### 3.3. Responder Analysis

Table 3 shows rates of treatment responders in each group for functional impairment and pain intensity at each follow-up point. There were no significant differences in the rate of MICDs between the two groups.

### 3.4. Subjective Satisfaction Scale

**Figure 4:**
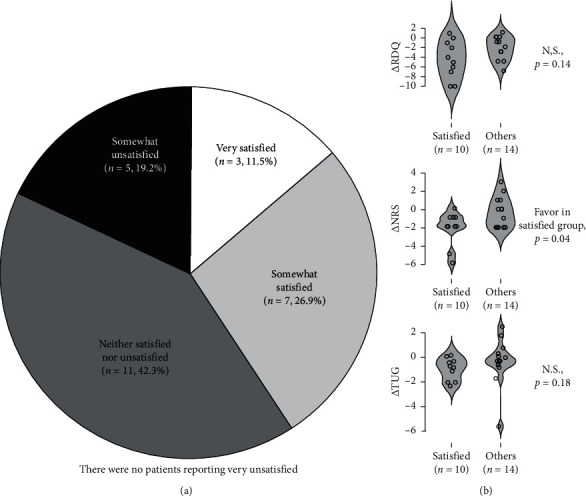
(a) Satisfaction rating by participants in the TAU + OLP group. (b) Difference in outcomes at week 12 between satisfied (Very Satisfied and Somewhat Satisfied) and other (Neither Satisfied nor Unsatisfied and Somewhat Unsatisfied) group. Outcome values are changes from the baseline value at week 12.

Figure 4 shows the findings of the subjective satisfaction assessment for patients in the OLP + TAU group at week 3 (*n* = 2) and week 12 (*n* = 24). Two patients in this group reported that they were not willing to continue the trial after week 3, and their satisfaction ratings were “Neither Satisfied nor Unsatisfied” and “Somewhat Unsatisfied,” respectively.

The number of patients reporting “Very Satisfied” and “Somewhat Satisfied” reached approximately 40% out of the allocated participants (Figure 4(a)). A further analysis showed that patients (*n* = 10) reporting “Satisfied” significantly improved in the pain-NRS score (*p* = 0.04) compared with those (*n* = 14) reporting “Neither Satisfied nor Unsatisfied” or “Somewhat Unsatisfied” (Figure 4(b)) at week 12.

## 4. Discussion

The present study investigated whether the OLP + TAU treatment was effective for Japanese patients with CLBP under the assumption that it would show the same efficacy as in previous reports. Our results revealed that OLP + TAU offered no significant superior benefit compared with TAU alone after 3- and 12-week interventions for Japanese patients with CLBP although significant improvements in functional outcome was obtained in both groups. It is important to note the difference between our results and the results of the previous two studies.

The two previous studies reported that patients with CLBP receiving OLP + TAU treatment showed significant reductions in pain intensity and disability scores at 3 weeks, with medium or greater effect sizes, compared with those receiving TAU only [8, 9]. Although these studies provided evidence that a nondeceptive placebo would be effective in patients with CLBP, the levels of pain reduction in the OLP + TAU group as well as the changes in the clinical outcomes in the TAU group merit further discussion.

In terms of changes in pain intensity after adjusting pain scores on a 0–10 pain scale, the magnitudes of pain reductions after a 3-week intervention (mean ± SD) were Δ1.5 ± 1.8 at the usual level in the study by Carvalho et al. and Δ0.6 ± 0.2 at the average level in the study by Kleine-Borgmann et al. [8, 9]. In contrast, we observed a pain reduction of Δ0.9 ± 1.8 at the present level in the OLP + TAU group at 3 weeks. Interestingly, the level of pain reduction in the OLP + TAU in our study was comparable with those reported by Carvalho et al. and Kleine-Borgmann et al. A recent editorial argues that a score of 0.7 out of 10 on the pain scale, which is below a clinically significant threshold for improvement, indicates no therapeutic value [25], and the accepted threshold for a clinically worthwhile effect was reported to be 2 points or more on a 10-point scale [23, 26].

Chronic pain is known to be influenced by different physical, psychological, and social factors known as “contextual factors” [27]. Contextual effects would not work in the previous two studies because they showed no improvements of clinical outcomes in the TAU group [8, 9]. On the other hand, contextual effects in chronic pain conditions predominantly include the placebo response and the regression to the mean phenomenon [28]. In this regard, participants in the TAU group showed significant improvements (time-course effects) in pain disability, indicating contextual effects in the current study.

A recent article argues what is usual care for low back pain [11]. Although there are a number of standard options for treating low back pain, modalities or agents that could be considered better are yet unknown. Blease et al. reported that controls should ideally be structurally equivalent conditions when compared with the active treatment, OLP [10]. In order to address this concern, we adjusted structural treatment conditions related to the format of treatment and the quality of the interactions by ensuring treatment by the same physician with the same treatment strategy (i.e., TAU), with the only difference being administration of OLP capsules.

Apart from the above issues, responsiveness to OLP may differ by race or culture [13, 14, 29]. A recent study has indicated that white Americans reported greater conditioning effects, reinforced relief expectations, and placebo effects when compared with black African Americans [14]. African Americans, Asians, and Hispanics have been reported to show higher pain sensitivity than non-Hispanic whites [30], and non-Caucasians were more prone to developing long-term chronic pain than Caucasians [31]. Racial differences in the responsiveness to OLP may explain the differences in the results between the present study and the two previous studies.

Although this study did not show superior clinical benefits of OLP treatment, we found that approximately 40% of the participants in the OLP + TAU group were at least somewhat satisfied with the treatment. A further analysis suggested that treatment satisfaction was associated with pain reduction in this group. These results imply that participant's preference or attitude may influence on treatment outcome in an open-placebo trial.

There are several limitations of the current study. First, assessments of outcomes were not blinded at all points, and all participants were treated by the same experimenter. Although this trial enabled structural equivalence of treatment between the two groups except OLP, there was a bias concerning of nonblind treatment by the experimenter. Blind allocation and treatment of OLP may solve this concern. Second, we did not assess expectations of OLP efficacy in the participants. Believes toward OLP efficacy could contribute to outcomes among this population. Third, only a small number of patients from a single medical center were included. A small sample size has to be reconsidered when there is a small difference between groups. The lack of a statistically significant difference in outcomes between the two groups in this study does not negate the clinical significance of OLP [32] since it could be attributed to a smaller-than-expected effect size. Given the small effect size (0.24) obtained by this study, a sample size of 432 (216 per each group) for a power of 0.80 with a significance level of *p* < 0.05 would be needed to detect a difference between the two groups. Further studies are required in this regard.

## 5. Conclusions

The present study investigated whether the OLP + TAU treatment was effective for Japanese patients with CLBP under the assumption of equal efficacy as in the previous reports. Our results revealed that OLP + TAU showed no significant superior benefit in comparison with TAU alone after 3- and 12-week interventions for Japanese patients with CLBP although significant improvements in functional disability was obtained in both groups at week 12.

## Figures and Tables

**Table 1:** Comparision of parameters at the baseline TAU group and TAU + OLP group

	TAU (*n* = 26)	OLP + TAU (*n* = 26)	Comparison (*p* value)
Female, *n* (%)	15 (57.7)	17 (65.4)	0.78
Age (years)	65.3 (13.8)	68.2 (13.0)	0.38
BMI (kg.m^2^)	22.4 (4.0)	24.1 (6.4)	0.55
RMDQ (range: 0–24)	10.3 (4.0)	9.9 (3.7)	0.72
NRS (range: 3–10)	5.5 (1.6)	5.3 (1.9)	0.48
TUG (sec)	9.1 (2.1)	8.5 (2.0)	0.27
History of lumber surgery, *n* (%)	7 (26.9%)	11 (42.3%)	0.38
Pain duration			0.41
6 months–1 year	8 (30.8%)	4 (15.4%)	
1–5 years	12 (46.2%)	14 (53.8%)	
>5 years	6 (23.1%)	8 (30.8%)	
Use of analgesic drugs, *n* (%)	17 (65.4%)	16 (61.5%)	1.0
NSAID oral	9 (34.6%)	5 (19.2%)	
NSAID transdermal	5 (19.2%)	4 (15.4%)	
Acetaminophen	3 (11.5%)	2 (7.7%)	
Pregabalin	5 (19.2%)	4 (15.4%)	
Duloxetine	0	0	
Weak opioid	2 (7.7%)	2 (7.7%)	
Strong opioid	0	0	
An extract^#^	0	1 (3.8%)	
Others	4 (15.4%)	2 (7.7%)	

^#^ An extract: an extract from inflamed cutaneous tissue of rabbits inoculated with vaccinia virus.

**Table 2:** Changes in outcomes at week 3 and week 12

		Changes from baseline (*n* = 26)	Changes from baseline (*n* = 24)
		Week 3	Week 12
RMDQ (range: 0–24)	TAU	−1.4 (3.6), 1.0 [−3.0, 0.0]	−2.3 (3.2), −2.0 [−2.3, −0.8]
	TAU + OLP	−2.2 (2.9), −1.0 [−4.0, 0.0]	−3.3 (3.2), −3.0 [−5.0, −0.8]

NRS (range: 0–10)	TAU	−0.2 (1.8), 0 [−1.8, 1.0]	−0.8 (1.9), −1 [−2.0, 1.0]
	TAU + OLP	−0.9 (1.8), −1.0 [−1.0, 0.0]	−1.1 (1.9), −1.0 [−2.0, 0.0]

TUG (sec)	TAU	−0.7 (1.5), −0.5 [−1.4, −0.1]	−1.1 (1.1), −1.0 [−1.7, −0.3]
	TAU + OLP	−0.7 (1.0), −0.4 [−1.0, −0.2]	−0.6 (1.5), −0.4 [−1.0, 0.0]

Data are present as mean (standard deviation) and median (interquartile range).

**Table 3:** Responders at week 3 and week 12.

		Number of responders (%)		Number of responders (%)	
		Week 3 (*n* = 26)	*p* value	Week 12 (*n* = 24)	*p* value
RMDQ (∆ ≤ −2)	TAU	11 (42.3%)	1.00	13 (54.2%)	0.56
	TAU + OLP	11 (42.3%)		15 (62.5%)	

NRS (∆ ≤ −2)	TAU	7 (26.9%)	0.51	7 (29.2%)	0.23
	TAU + OLP	5 (19.2%)		11 (45.8%)	

A responder is defined as one that shows changes of at least 2-point from the baseline value at each follow-up point.

## Data Availability

The data used to support the findings of this study are available from the corresponding author upon request.
